# Manual Muscle Testing of the Scapula and the Upper Limb through Bedside Examination

**DOI:** 10.21315/mjms2023.30.1.17

**Published:** 2023-02-28

**Authors:** Kugan Vijian, Yap Teck Cheng, Zamzuri Idris, Abdul Rahman Izaini Ghani, Sanihah Abdul Halim, Jafri Malin Abdullah

**Affiliations:** 1 Department of Neurosciences, School of Medical Sciences, Universiti Sains Malaysia, Kelantan, Malaysia; 2 Hospital Universiti Sains Malaysia, Universiti Sains Malaysia, Kelantan, Malaysia; 3 Department of Neurosurgery, Hospital Umum Sarawak, Sarawak, Malaysia; 4 Department of Neurosurgery, Hospital Queen Elizabeth, Sabah, Malaysia; 5 Neurology Unit, Department of Internal Medicine, School of Medical Sciences, Universiti Sains Malaysia, Kelantan, Malaysia; 6 Brain and Behaviour Cluster, School of Medical Sciences, Universiti Sains Malaysia, Kelantan, Malaysia

**Keywords:** manual muscle testing, upper limbs, scapula

## Abstract

Neurological examination is an important tool in diagnosing patients with neurological and neurosurgical conditions. As the complexity and knowledge of neurological and neurosurgical conditions increases, we are now required to learn and indoctrinate our peers and students with the correct skills and methods of examination. Emphasis on the correct techniques of testing muscle strength is essential to avoid errors in recording muscle power and in testing specific muscles which may have overlapping functions. The manual muscle testing of muscles of scapula and upper limbs was performed as to mimic a bedside clinical examination and involved an examiner, a patient and a videographer. The manual muscle testing has been performed in rostrocaudal manner starting from the scapula and ending with the thumbs. A reliable and consistent method of manual muscle testing is lacking among students and clinicians. By adhering to the methods delineated in our text and accompanying video, we hope to reduce inter-examiner variability and increase the reliability and validity of this important examination.

## Introduction

Neurological examination is an important tool for diagnosing patients with neurological and neurosurgical conditions. Introduced in the 1800s and refined over the years by figures such as Wilhelm Erb and Joseph Babinski (1), this assessment method is being taught during early medical education with the goal of creating competent examiners of the neurological system. The examination spans from the evaluation of higher mental functions to motor and sensory systems. Assessment of the motor system is arguably one of the most integral parts of the examination, as it can reveal the possible side and location of an intracranial or spinal lesion. Motor examination comprises inspection of the limbs and muscles, assessment of muscle tone, power, reflexes and plantar response. The patients’ gait and cerebellar function are also assessed to complete the examination.

Emphasis on the correct techniques for testing muscle strength is needed to avoid errors in recording muscle power and to test specific muscles that may have overlapping functions. Although the definitions and methods of grading muscle power in different muscle groups may differ, a standardised technique should be followed to avoid discrepancies among examiners. The instructions given to the patient to elicit certain movements are also key for activating the muscle that needs to be tested. In particular, manual muscle testing is a simple assessment method that does not require expensive tools or equipment. Reliable knowledge of the tested muscles anatomy and specific movements is usually sufficient for an accurate neurological examination.

As the complexity and knowledge of neurological and neurosurgical conditions increases, we are required to learn and to instruct our peers and students with the correct skills and methods of examination. Thus, this study aimed to delineate the correct methods for testing the muscles of the scapula, shoulder, elbow, forearm, wrist, hands, thumbs and fingers.

## Methods

### Setting

Manual muscle testing of the scapula and upper limb muscles was performed at the Student Resource Center of the Hospital Universiti Sains Malaysia (HUSM), Kubang Kerian. HUSM is a tertiary teaching university hospital in Kelantan.

### Equipment and Personnel

An examination was conducted mimicking bedside clinical assessment. This comprised a doctor and a patient. The examination was performed with the patient sitting or lying on an examination bed. A cardiac table was used for assessment of the forearm muscles and hands. A videographer was present throughout the examination to record the procedure and served as a way to identify any mistakes made during manual muscle testing.

### Video

A video recorded during the manual muscle testing was edited to incorporate slides explaining each examination method, followed by the examination itself. The videos are available in the following links.

Scapula examination:

https://youtu.be/22k_1B7J_2k

Shoulder examination:

https://youtu.be/H4-fdJN3MQk

Elbow examination:

https://youtu.be/TDHpUbvK-vw

Forearm examination:

https://youtu.be/OBp-BG2y3lQ

Wrist examination:

https://youtu.be/yw2DHPMF6YI

Fingers examination:

https://youtu.be/bfD_PRI3ko0

Thumb examination:

https://youtu.be/LY8pL7C9KFg

### Manual Muscle Testing

Manual muscle testing is performed in a rostrocaudal manner starting at the scapula and ending with the thumbs. The grading of the muscles is performed according to the Medical Research Council (MRC) (2) grading for muscle power, which defines the following categories:

0 = No contraction
1 = Flicker or trace of contraction
2 = Active movement, with gravity elimination
3 = Active movement against gravity
4 = Active movement against gravity and resistance
5 = Normal power

The testing of each muscle is further divided into examinations of its specific movements. For example, the possible movements of the scapula comprise abduction and upward rotation, elevation, adduction, depression and adduction, and adduction and downward rotation.

The primary muscle involved and tested is also mentioned for the accompanying movement.

### Scapula

The movements of the scapula include:

- abduction and upward rotation
- elevation
- adduction
- depression and adduction
- adduction and downward rotation

#### i) Abduction and upward rotation

For scapular abduction and upward rotation, the primary muscle tested is the serratus anterior.

##### Grading

To examine grades 5–3 of the scapular abduction and upward rotation, the patient is seated over the end of the table with hands on the lap while the examiner stands at the side of the patient with the hand putting resistance on the arm proximal to the elbow. The other hand uses the thumb and index finger as well as the web space to palpate the edges of the scapula at the inferior angle and along the vertebral and axillary borders.

The patient is instructed to raise the arm to approximately 130° of flexion, with the elbow extended. Under normal conditions, the scapula should rotate upwardly and abduct without winging.

Grade 5: The scapula sustains its abducted and rotated position against maximal resistance in the downwards direction.

Grade 4: The scapula has ‘give’ against maximal resistance but is able to hold strong-to-moderate resistance.

Grade 3: The scapula moves over the full range of motion without winging but can tolerate no resistance.

For grades 2 to 0, patient is in the sitting position with the arm flexed up to 130° and the elbow extended. The examiner supports the patient’s arm at the elbow, maintains it above the horizontal axis, and monitor scapular motion and muscle activity using a light grasp at the inferior angle of the scapula. The examiner must not resist the motion.

Grade 2: The scapula moves during attempt to hold in test position.

Grade 1: Contractile activity is detected without movement.

Grade 0: No palpable contractile activity.

#### ii) Elevation

For scapular elevation, the muscles tested are the trapezius and levator scapulae.

##### Grading

For testing grades 5–3 of scapular elevation, the patient is seated at the end or side of the table. The examiner stands behind the patient with hands contoured over the top of both shoulders, providing resistance in a downward direction.

The patient is then instructed to elevate or shrug the shoulder. The test can be performed simultaneously on both sides.

Grade 5: Patient can shrug the shoulder and hold against the maximal resistance.

Grade 4: Patient can shrug the shoulder and hold against strong-to-moderate resistance. The shoulder may have ‘give’ at the endpoint.

Grade 3: Patient can complete the full range of motion without resistance.

For grades 2–0, the patient is in the prone position with the head turned away from the test side. The examiner supports the anterior shoulder and palpates the upper trapezius muscle.

Grade 2: Scapula moves through the full range in a gravity-eliminated manner.

Grade 1: Contractile activity is detected without movement.

Grade 0: No palpable contractile activity.

#### iii) Adduction

For scapular adduction, the middle fibers of the trapezius and rhomboid major are tested.

##### Grading

For testing grades 5–3 of scapular adduction, the patient is in the prone position with the shoulder at the edge of the table. The shoulder is abducted to 90° and the elbow flexed at tight angle. The head is turned to either side for comfort.

The examiner stands on the test side close to the arm of the patient, stabilising the contralateral scapular area to prevent trunk rotation. The examiner applies force downwards toward the floor. The fingers of the other hand can palpate the middle fibers of the trapezius at the spine of the scapula, from the acromion to the vertebral column. The patient is instructed to abduct the arm horizontally and adduct the scapula.

Grade 5: Patient can complete available scapular adduction and hold the end position against maximal resistance.

Grade 4: Patient tolerates strong-to-moderate resistance.

Grade 3: Patient can complete available range, but without resistance.

For grades 2–0, the patient is positioned similarly. The examiner uses one hand to support the patient’s shoulder and arm and another hand to palpate the muscle. The patient is instructed to perform the same procedure as that for grades 5 to 3.

Grade 2: patient can complete the full range of motion without the weight of the arm.

Grade 1: contractile activity is detected without movement.

Grade 0: no palpable contractile activity.

#### iv) Depression and adduction

The muscles tested for scapular depression and abduction include the middle and lower fibres of trapezius.

##### Grading

For testing grades 5–3 of scapular depression and abduction, the patient is in the prone position with test arm over head to approximately 145° of abduction (in line with lower fibres of the trapezius), the forearm in the neutral position with the thumb pointing towards the ceiling. The head can be turned to either side for comfort. The examiner stands on the test side with the hand providing resistance just proximal to the elbow towards the floor. The fingers of the other hand palpate below the spine of the scapula and across the thoracic vertebrae, following the muscle as it curves down to the lower thoracic vertebrae.

The patient is then instructed to raise the arm from the table at least at the ear level and hold against resistance.

Grade 5: Patient can complete available range and hold it against maximal resistance.

Grade 4: Patient can tolerate strong-to-moderate resistance.

Grade 3: Patient can complete available range of motion but without resistance.

For grades 2–0, the patient is positioned the same as that for grades 5–3, but the examiner supports the arm below the elbow. The patient is instructed to attempt to lift the arm from the table.

Grade 2: Patient can complete the range of motion without the weight of the arm.

Grade 1: Contractile activity is detected without movement.

Grade 0: No palpable contractile activity.

#### v) Adduction and downward rotation

The muscles tested for scapular adduction and downward rotation are the rhomboid major and rhomboid minor.

##### Grading

For testing grades 5–3, the patient is positioned in the prone position with the shoulder internally rotated and the arm adducted across the back with the elbow flexed and the hand resting on the back.

The examiner stands at the test side with the hand used for resistance places on the humerus just above the elbow, and resistance is given in at downwards and outwards directions. The patient is asked to lift the hand off the back and maintain the arm position across the back while the examiner applies resistance over the elbow.

Grade 5: Patient can complete the range of motion and hold against maximal resistance.

Grade 4: Patient can complete the range and hold against strong-to-moderate resistance.

Grade 3: Patient can complete range, but without resistance.

For testing grades 2–0, the patient is positioned in the sitting position with the shoulder internally rotated and the arm extended and adducted behind the back.

The examiner supports the arm by grasping the wrist, and the other hand palpates the muscles under the vertebral border of the scapula. The patient is instructed to lift the hand from the back.

Grade 2: Completes full range of motion with eliminated gravity force.

Grade 1: Contractile activity detected without movement.

Grade 0: No palpable contractile activity.

### Shoulder

The movements of the shoulder include:

- Flexion
- Extension
- Abduction
- Horizontal abduction
- Horizontal adduction
- External rotation
- Internal rotation

#### i) Flexion

The muscles tested for shoulder flexion include the anterior deltoid and coracobrachialis.

##### Grading

The patient is in the sitting position with the arms on the side, elbow slightly flexed and forearm pronated.

The examiner stands at the test side with the hand providing resistance contoured over the distal humerus, and the other hand stabilises the shoulder.

Patient is then instructed to flex the shoulder to 90° without rotation or horizontal movement. The scapula should be allowed to abduct and rotate upwards.

Grade 5: Patient can hold the end position against maximal resistance.

Grade 4: Patient can hold against strong-to-moderate resistance.

Grade 3: Patient can complete test range of motion up to 90° without resistance.

For testing grades 2–0, the patient is seated with the arm at the sides and the elbow slightly flexed. The examiner stands on the test side with the fingers used for palpation placed over the superior and anterior surfaces of the deltoid. Patient is instructed to flex the shoulder up to 90°.

Grade 2: Patient can complete partial test range as this is against gravity.

Grade 1: Contractile activity detected without movement.

Grade 0: Co palpable contractile activity.

#### ii) Extension

The muscles tested for shoulder extension are the latissimus dorsi, posterior deltoid, and teres major.

##### Grading

For grades 5–2, the patient is positioned supine with arms at the side and the shoulder internally rotated with the palm up.

The examiner stands on the test side, and the hand used for resistance is contoured over the posterior arm just proximal to the elbow. The patient is instructed to raise the arm off the table and keep his elbow straight.

Grade 5: Patient can complete available range and hold against maximal resistance.

Grade 4: Patient can complete the available range and hold against strong-to-moderate resistance.

Grade 3: Patient can complete available range, but without resistance.

Grade 2: Patient able to complete partial range of motion.

For grades 1 and 0, the patient is positioned similarly, but the examiner places the finger for palpation on the side of the thoracic wall below and lateral to the inferior angle of the scapula. Palpates over the posterior shoulder just superior to the axilla for posterior deltoid fibre. The teres major is palpated on the lateral border of the scapula just below the axilla. the patient attempts to lift the arm from the table on request.

Grade 1: Contractile activity detected without movement.

Grade 0: No palpable contractile activity.

#### iii) Abduction

The muscles tested for shoulder abduction are the middle fibers of the deltoid and supraspinatus.

##### Grading

For testing grades 5–3, the patient is seated with the arm at the side and the elbow slightly flexed. The examiner stands behind the patient with the hand-giving resistance contoured over the arm just proximal to the elbow and applies forces downwards to the floor. The patient is instructed to abduct the arm at 90°.

Grade 5: Patient can hold end test position against maximal resistance.

Grade 4: Patient can hold end test position against strong-to-moderate resistance.

Grade 3: Patient can complete range of motion with no manual resistance.

For testing grades 2–0, the patient is positioned supine with the arm at the side supported on the table. The examiner stands on the test side with the hand used for palpation and is positioned at the deltoid lateral to the acromial process on the superior aspect of the shoulder. The supraspinatus can be palpated by placing the fingers deep under the trapezius, in the supraspinous fossa of the scapula. The patient is instructed to abduct the shoulder by sliding the arm on the table.

Grade 2: Complete full range of motion in a gravity-eliminated manner.

Grade 1: Contractile activity detected without movement.

Grade 0: No palpable contractile activity.

#### iv) Horizontal abduction

The muscle tested for horizontal abduction of the shoulder is the posterior fibers of the deltoid. It originates in the spine on the lower lip of the crest of the scapula and is inserted into the deltoid tuberosity of the humerus.

##### Grading

For grades 5–3, the patient is positioned in prone with shoulder abducted to 90° and the forearm off edge of table with the elbow flexed. The examiner stands at the test side with the hand-giving resistance contoured over the posterior arm just proximal to the elbow. The patient is instructed to abduct the shoulder horizontally against resistance.

Grade 5: Completes the full range of motion and hold the end position against maximal resistance.

Grade 4: Completes the range of motion and holds the end position against strong-to-moderate resistance.

Grade 3: Completes range of motion with no manual resistance.

For grading 2–0, the patient is positioned sitting with the arm supported on table at 90° of abduction and the elbow partially flexed. The examiner stands behind the patient, stabilised by contouring one hand over the scapula, and palpates the fibers of the posterior deltoid below and lateral to the spine of the scapula and on the posterior aspect of the proximal arm adjacent to the axilla. The patient is instructed to slide the arm across the table in horizontal abduction.

Grade 2: Moves through full range of motion.

Grade 1: Contractile activity detected without movement.

Grade 0: No palpable contractile activity.

#### v) Horizontal adduction

The muscle tested for shoulder horizontal adduction is the pectoralis major.

For grades 5–3, the patient is positioned supine with the shoulder abducted to 90° and the elbow flexed to 90°.

The examiner stands at the side of the tested shoulder with the hand used for resistance contoured proximal to the wrist, and the other hand is used to check the activity of the pectoralis major on the upper aspect of the chest just medial to the shoulder joint. There are three manners of testing the shoulder horizontal adduction. When the whole muscle is tested, the patient is instructed to adduct the shoulder horizontally through the available range of motion.

When testing the clavicular head, the patient’s motion begins at 60° abduction and moves upward and inward across the body. The examiner applies resistance above the wrist in a downward and outward direction. The examiner then palpates the clavicular fibres of the pectoralis major under the medial half of the clavicle.

To test the sternal head, motion begins at 120° abduction and moves diagonally downward and inward towards the patient’s opposite hip. The examiner applies resistance above the wrist in the upward and outward directions. The examiner palpates the sternal head on the chest wall at the lower anterior border of the axilla.

Grade 5: Complete range of motion and takes maximal resistance.

Grade 4: Complete range of motion and takes strong-to-moderate resistance.

Grade 3: Complete range of motion in all three tests with no resistance other than the weight of the extremity.

Testing grades 2–0: the patient is in the sitting position with test arm supported on the table at the level of axilla and arm in 90° abduction and elbow slightly flexed. The friction with the table surface should be minimised. The examiner stands behind the patient and palpates the pectoralis muscle on the anterior aspect of the chest medial to the shoulder joint. The patient is instructed to move across the table.

Grade 2: Adduct the shoulder horizontally through the available range of motion with the weight of the arm supported by the table.

Grade 1: Contractile activity detected without movement.

Grade 0: No palpable contractile activity.

#### vi) External rotation

The muscles tested for shoulder external rotation are the infraspinatus and teres minor.

##### Grading

To test grades 5–3, the patient is positioned prone with the head turned towards the tested side, the shoulder abducted to 90°, and the arm fully supported on the table; the forearm hanging vertically over the edge of the table. The examiner stands on the test side at the level of the patient’s waist. Two fingers of one hand are used to provide resistance, and the other hand supports the elbow to provide counter pressure at the end of the range. The patient is then instructed to move the forearm upward through the range of external rotation.

Grade 5: Completes the available range of motion and holds firmly against two-finger resistance.

Grade 4: Completes the available range but yields or gives away at the end of the range. Able to hold strong-to-moderate resistance.

Grade 3: Completes available range, but unable to tolerate manual resistance.

For grading 2–0, the patient is in the prone position and the head is turned to the tested side and trunk at the edge of the table. The entire limb hangs down loosely from the shoulder in the neutral rotation and on a palm-facing table. The examiner stands or sits on a low stool on the test side of the patient at the shoulder level and palpates the infraspinatus over the body of the scapula below the spine in the infraspinous fossa. The examiner palpates the teres minor at the inferior margin of the axilla and along the axillary border of the scapula. The patient is then instructed to rotate the shoulder externally.

Grade 2: Completes available range in a gravity-eliminated position.

Grade 1: Contractile activity detected without movement.

Grade 0: No palpable contractile activity.

#### vii) Internal rotation

The muscles tested for shoulder internal rotation include the subscapularis, pectoralis major (clavicular and sternal head) and latissimus dorsi.

##### Grading

For grades 5–3, the patient is in the prone position with the head turned towards the test side. The shoulder is abducted to 90°, and forearm hanging over the edge of the table. The examiner stands on the test side with the hand-giving resistance placed on the volar side of the forearm just above the wrist, and the other hand provides a counterforce at the elbow. The examiner applies resistance in the downward and forward directions, and the counterforce is applied backwards and upwards. The patient is instructed to move their arm through the available range of internal rotation (backward and upward).

Grade 5: Completes available range and holds firmly against maximal resistance.

Grade 4: Completes the available range, but there is a ‘spongy’ feeling against strong resistance or being able to hold against strong-to-moderate resistance.

Grade 3: Completes the range of motion without any manual resistance.

For grades 2–0, the patient is in the prone and similar position as that used for grade 5. The examiner stands on the test side or sits on the low stool, and the hand used for palpation must find the subscapularis tendon deep in the central area of the axilla. The patient is then instructed to internally rotate the arm with the thumb so that the palm faces outward or away from the table.

Grade 2: Completes available range.

Grade 1: Contractile activity detected without movement.

Grade 0: No palpable contractile activity.

### Elbow

The movements of the elbow include:

- Flexion
- Extension

#### i) Flexion

The muscles tested for elbow flexion include the biceps brachii, brachialis, and brachioradialis.

##### Grading

For grades 5–3, the patient is positioned in the sitting position with the arms at the sides. If the biceps brachii are checked, the forearm is supinated. To check the brachialis, the forearm is pronated. To check the brachioradialis, the forearm is placed in the mid-position or neutral position. The examiner stands in front of the patient, and the hand providing resistance is contoured over the flexor surface of the forearm proximal to the wrist. In contrast, a counterforce is applied by cupping the palm over the anterior surface of the shoulder. The patient is instructed to flex the elbow through the range of motion for all three tests.

Grade 5: Completes range and holds firmly against maximal resistance.

Grade 4: Completes the range against strong-to-moderate resistance.

Grade 3: Completes the range of motion with each forearm position with no manual resistance.

For grade 2, the patient is in the sitting position with the arm abducted to 90° and supported by an examiner. Again, the forearm is supinated for the biceps, pronated for the brachialis, and in a neutral position for the brachioradialis. The examiner stands in front of the patient and supports the abducted arm under the elbow and wrist, and then palpates the tendon of the biceps. The patient is instructed to flex the elbow.

Grade 2: Completes the range of motion in all three tests in a gravity-eliminated manner.

For grading from 1–0, the patient is in the supine position and the examiner stands on the test side. The patient attempts to flex the elbow with the forearm supinated, pronated, and in neutral position.

Grade 1: Contractile activity detected without movement.

Grade 0: No palpable contractile activity.

#### ii) Extension

The muscle tested for elbow extension is the triceps brachii, which has three heads.

##### Grading

For grades 5–3, the patient is prone, starting with the arm in 90° abduction and the forearm flexed and hanging vertically over the side of table. The examiner supports just above the elbow, and another hand is used to apply downwards resistance to the dorsal surface of the forearm. The patient is instructed to extend the elbow until the forearm is parallel to the floor.

Grade 5: Completes available range of motion and holds firmly against maximal resistance.

Grade 4: Completes the available range of motion and firmly holds against strong-to-moderate resistance.

Grade 3: Completes the range of motion with no manual resistance.

For grades 2–0, the patient is in the sitting position, with the arm abducted to 90°, the shoulder in neutral rotation, and the elbow flexed at approximately 45°. The examiner stands on the test side and supports the limb at the elbow. The patient attempts to extend the elbow.

Grade 2: Completes the range of motion in the absence of gravity.

Grade 1: Contractile activity detected without movement. The examiner can feel tension in the triceps tendon immediately proximal to the olecranon.

Grade 0: No palpable contractile activity.

### Forearm

The movements of the forearm include:

- Supination
- Pronation

#### i) Forearm supination

The muscles tested are the biceps brachii and supinator.

##### Grading

The patient is tested in the sitting position with the elbow flexed. The examiner applies force into forearm pronation.

Grade 5: Holds against maximal resistance.

Grade 4: Holds against strong-to-moderate resistance.

The examiner observes for range of motion while stabilising the arm.

Grade 3: Completes the full range of motion without resistance.

The patient is placed in the sitting position with the elbow flexed. The examiner palpates the appropriate muscle and supports the arm.

Grade 2: Completes the partial range of motion.

Grade 1: Contractile activity is detected without movement.

Grade 0: No palpable contractile activity.

#### ii) Forearm pronation

The muscles tested are the pronator teres and pronator quadratus.

##### Grading

The patient is placed in the sitting position with the elbow flexed. The examiner then applies force into forearm supination.

Grade 5: Holds against maximal resistance.

Grade 4: Holds against strong-to-moderate resistance.

The examiner observes for range of motion while stabilising the arm.

Grade 3: Completes full range of motion without resistance.

The patient is placed in the sitting position with the elbow flexed. The examiner palpates the appropriate muscle and supports the arm.

Grade 2: Completes the partial range of motion.

Grade 1: Contractile activity is detected without movement.

Grade 0: No palpable contractile activity.

### Wrist

The movements of the wrist include:

- Flexion
- Extension

#### i) Wrist flexion

The muscles tested are the flexor carpi radialis and flexor carpi ulnaris.

##### Grading

The patient is in the sitting position with the forearm supinated. The examiner then applies force into wrist extension.

Grade 5: Holds against maximal resistance.

Grade 4: Holds against strong-to-moderate resistance.

Grade 3: Completes movement, holds without resistance.

The patient is seated with the forearm in the neutral position. The examiner then checks the range of motion and stabilises the forearm.

Grade 2: Completes the full range of motion with gravity minimised.

The patient is in the sitting position with the forearm supinated. The examiner palpates the flexor muscles and tendons.

Grade 1: Contractile activity is detected without movement.

Grade 0: No palpable contractile activity.

#### ii) Wrist extension

The muscles tested are the extensor carpi radialis longus, brevis, and extensor carpi ulnaris.

##### Grading

The patient is in the sitting position with the forearm pronated. The examiner applies force into wrist flexion.

Grade 5: Holds against maximal resistance.

Grade 4: Holds against strong-to-moderate resistance.

Grade 3: Completes movement, holds without resistance.

The patient is seated with the forearm in the neutral position. The examiner checks the range of motion and stabilised the forearm.

Grade 2: Completes the full range of motion with gravity minimised.

With the patient in the sitting position, with the forearm pronated, the examiner palpates the extensor muscles and tendons.

Grade 1: Contractile activity detected without movement.

Grade 0: No palpable contractile activity.

### Finger

The movements of the fingers include:

- Finger metacarpophalageal joint flexion
- Finger proximal interphalangeal joint flexion
- Finger distal interphalangeal joint flexion
- Finger metacarpophalangeal joint extension
- Finger abduction
- Finger adduction

#### i) Finger metacarpophalageal (MCP) joint flexion

The muscles tested are the lumbricals, dorsal interossei, and palmar interossei.

##### Grading

The patient is placed in the sitting position, with the forearm supinated. The examiner applies force into MCP extension.

Grades 5 and 4: Holds against strong-to-moderate resistance.

However, it is difficult to differentiate grade 5 and 4 in finger movements.

Grade 3: Completes full range of motion without resistance.

With the patient in the sitting position with the forearm neutral, the examiner stabilises the metacarpals.

Grade 2: Completes full range of motion.

Grade 1: Minimal motion/palpation for contractile activity (difficult).

Grade 0: No palpable contractile activity.

#### ii) Finger proximal interphalangeal (PIP) joint flexion

The muscle tested is the flexor digitorum superficialis.

##### Grading

The patient is in the sitting position with the forearm supinated. The examiner applies force into PIP extension.

Grades 5 and 4: Patient is able to hold against strong-to-moderate resistance.

Grade 3: Completes full range of motion without resistance.

The patient is seated with the forearm neutral and the examiner palpates the flexor muscle tendons.

Grade 2: Completes the full range of motion.

Grade 1: Minimal motion/palpation for contractile activity (difficult).

Grade 0: No palpable contractile activity.

#### iii) Finger distal interphalangeal (DIP) joint flexion

The muscles tested are the flexor digitorum profundus.

##### Grading

The patient is in the sitting position with the forearm supinated. The examiner then applies force into DIP extension.

Grades 5 and 4: Holds against strong-to-moderate resistance.

Grade 3: Completes the full range of motion without resistance.

The examiner palpates the flexor muscle tendons with the patient in the sitting position with the forearm neutral.

Grade 2: Completes the full range of motion.

Grade 1: Minimal motion/palpation for contractile activity (difficult).

Grade 0: No palpable contractile activity.

#### iv) Finger metacarpophalangeal joint extension

The muscles tested are the extensor digitorum, extensor indices, and the extensor digiti minimi.

##### Grading

The patient is in the sitting position with the forearm pronated. The examiner applies force into MCP flexion.

Grades 5 and 4: Holds against strong-to-moderate resistance.

Grade 3: Completes the full range of motion without resistance.

The examiner palpates the extensor muscle tendons with the patient in the sitting position, with the forearm neutral.

Grade 2: Completes the full range of motion.

Grade 1: Visible minimal motion/ palpation for contractile activity (difficult).

Grade 0: No palpable contractile activity.

#### v) Finger abduction

The muscles tested are the dorsal interossei and abductor digiti minimi.

##### Grading

The patient is placed in the sitting position with the forearm pronated. The examiner applies force and pinches the pairs of fingers into adduction.

Grades 5 and 4: Holds against strong-to-moderate resistance.

With the patient in the sitting position, with the forearm pronated, the examiner observes the amount of motion.

Grade 3: Completes the full range of motion for any given finger.

Grade 2: Completes the partial range of motion for any given finger.

Grades 1 and 0: Not assigned for this test.

#### vi) Finger adduction

The muscle tested is the palmar interossei.

##### Grading

The patient is in the sitting position with the forearm pronated. The examiner applies force and pulls the fingers into abduction.

Grades 5 and 4: Holds against strong-to-moderate resistance.

With the patient in the sitting position and the forearm pronated, the examiner observes the amount of motion.

Grade 3: Completes the full range of motion for any given finger.

Grade 2: Completes the partial range of motion for any given finger.

Grades 1 and 0: Not assigned for this test.

### Thumb

The movements of the thumb include:

- Thumb metacarpophalangeal and interphalangeal flexion
- Thumb metacarpophalangeal and interphalangeal extension
- Thumb abduction
- Thumb adduction
- Thumb and little finger opposition

#### i) Thumb metacarpophalangeal and interphalangeal (IP) flexion

The muscles tested are the flexor pollicis longus (FPL) and flexor pollicis brevis (FPB).

##### Grading

The patient is in the sitting position with the forearm supinated. The examiner applies force to the thumb extension. Force is applied to the proximal phalanx for the FPB and to the distal phalanx for the FPL.

Grades 5 and 4: Holds against strong-to-moderate resistance.

With the patient in the sitting position with the forearm supinated, the examiner observes the MCP and IP range of motion.

Grade 3: Completes full range of motion for each joint.

With the patient in the sitting position, with the forearm supinated, the examiner observes the amount of motion and palpates the appropriate tendon.

Grade 2: Completes the partial range of motion for joint being tested.

Grade 1: Palpable muscle activity without motion.

Grade 0: No palpable contractile activity.

#### ii) Thumb metacarpophalangeal and interphalangeal extension

The muscles tested are the extensor pollicis longus and extensor pollicis brevis.

##### Grading

The patient is seated with the forearm in neutral position. The examiner applies force to the flexion at the distal phalanx.

Grades 5 and 4: Holds against strong-to-moderate resistance.

With the patient in the sitting position with the forearm neutral, the examiner observes the MCP and IP range of motion.

Grade 3: Completes full range of motion.

With the patient in the sitting position with the forearm pronated, the examiner observes the amount of motion and palpates the tendon.

Grade 2: Completes full range of motion with gravity minimised.

Grade 1: Palpable muscle activity without motion.

Grade 0: No palpable contractile activity.

#### iii) Thumb abduction

The muscles tested are the abductor pollicis longus (APL) and abductor pollicis brevis (APB).

##### Grading

The patient is in the sitting position with the forearm supinated. The examiner applies force into adduction.

Force is applied to the distal end of 1st metacarpal for APL and to the proximal phalanx for APB.

Grade 5: Holds against maximum finger resistance.

Grade 4: Holds against moderate finger resistance.

With the patient in the sitting position with the forearm supinated, the examiner observed the range of motion.

Grade 3: Completes full range of motion.

With the patient in the sitting position with the forearm supinated. The examiner observes the amount of motion and palpated the appropriate tendon.

Grade 2: Completes partial range of motion.

Grade 1: Palpable muscle activity without motion.

Grade 0: No palpable contractile activity.

#### iv) Thumb adduction

The muscle tested is the adductor pollicis.

##### Grading

The patient is in the sitting position with the forearm pronated. The examiner applies force to abduction of the thumb.

Grade 5: Holds against maximum finger resistance.

Grade 4: Yields slightly against strong finger resistance.

Grade 3: Complete full range of motion without resistance.

With the patient in the sitting position with the forearm neutral, the examiner observes the amount of motion.

Grade 2: Completes full range of motion with gravity minimised.

With the patient in the sitting position with the forearm neutral, the examiner palpates the adductor pollicis in the web space.

Grade 1: Palpable muscle activity without motion.

Grade 0: No palpable contractile activity.

#### v) Thumb and little finger opposition

The muscles tested are the opponens pollicis and opponens digiti minimi.

##### Grading

The patient is in the sitting position with the forearm supinated. The examiner applies force to separate the thenar and hypothenar eminences. The force is applied to first metacarpal for opponens pollicis and applied to fifth metacarpal for opponens digiti minimi.

Grade 5: Holds against maximum resistance.

Grade 4: Holds against moderate resistance.

With the patient in the sitting position, with the forearm supinated, the examiner observes the amount of motion.

Grade 3: Completes full range of motion.

With the patient in the sitting position with the forearm supinated, the examiner palpates both opponens muscles without restricting movement.

Grade 2: Completes partial range of motion.

Grade 1: Palpable muscle activity without motion.

Grade 0: No palpable contractile activity.

## Discussion

Despite being a common and frequently performed part of neurological examination, muscle testing may result in discrepancies among examiners, and even across different disciplines such as physiotherapy, rehabilitation and neurology. Manual muscle testing is the most common method for assessing muscle strength impairments (3). As outlined in the text above, we have described a standardised method for assessing muscle strength for movements of the scapula and upper limbs. The methods described are practical and suitable for bedside clinical examinations.

Based on a review of the reliability and validity of manual muscle testing (3), a list of factors during the examination has been presented. These factors include proper positioning to ensure that the muscle tested is the prime mover, adequate stabilisation, observation of the patient’s maintenance of the test position and performance of the test, consistency in timing and ensuring painless contacts. In our manual muscle testing method, the examination is performed without the use of any equipment. However, specialised equipment such as isokinetic machines and dynamometers are available for more objective muscle strength testing (4). This equipment is mainly utilised for research purposes and is not readily available for bedside usage. Walther (5) describes the importance of a competent examiner by stating, “Presently the best ‘instrument’ to perform manual muscle testing is a well-trained examiner, using his perception of time and force with knowledge of anatomy and physiology of muscle testing.”

Manual muscle testing is often used by clinicians to detect weakness in specific muscles, which may aid in identifying and localising lesions. Hence, sound knowledge of the anatomy, physiology and neurology of the neuromuscular system is necessary. In addition to being a clinical diagnostic tool, these examinations have been widely utilised by physiotherapists to evaluate patient progress after therapy (6). Applied kinesiology chiropractors use muscle testing to determine whether manipulable impairments in neurological function (controlling muscle function) are present prior to interventions.

## Conclusion

A reliable and consistent method describing manual muscle testing for students and clinicians is lacking. We have provided a textual description and accompanying videos of manual muscle testing of the movements of the scapula, shoulder, elbow, forearm, wrist, hands, thumbs and fingers. By adhering to the delineated methods, we hope to reduce inter-examiner variability and increase the reliability and validity of this important examination.
